# *GhBEE3-Like* gene regulated by brassinosteroids is involved in cotton drought tolerance

**DOI:** 10.3389/fpls.2022.1019146

**Published:** 2022-10-13

**Authors:** Eryong Chen, Xiaobei Yang, Ruie Liu, Mengke Zhang, Meng Zhang, Feng Zhou, Dongxiao Li, Haiyan Hu, Chengwei Li

**Affiliations:** ^1^ Henan Engineering Research Center of Crop Genome Editing, School of Life Science and Technology, Henan Institute of Science and Technology, Xinxiang, China; ^2^ International Joint Laboratory of Plant Genetic Improvement and Soil Remediation, School of Life Science and Technology, Henan Institute of Science and Technology, Xinxiang, China; ^3^ Shanghai Center for Plant Stress Biology, National Key Laboratory of Plant Molecular Genetics, Center for Excellence in Molecular Plant Sciences, Chinese Academy of Sciences, Shanghai, China; ^4^ College of Biological Engineering, Henan University of Technology, Zhengzhou, China

**Keywords:** *GhBEE3-Like*, bHLH transcription factor, brassinosteroids (BRs), GhBZR1, drought tolerance, cotton (*Gossypium hirsutum* L.)

## Abstract

Brassinosteroids (BRs) are important phytohormones that play a vital role in plant drought tolerance, but their mechanisms in cotton (*Gossypium hirsutum* L.) are poorly understood. Numerous basic helix-loop-helix (bHLH) family genes are involved in the responses to both BRs and drought stress. *GhBEE3-Like*, a bHLH transcription factor, is repressed by both 24-epi-BL (an active BR substance) and PEG8000 (drought simulation) treatments in cotton. Moreover, GhBZR1, a crucial transcription factor in BR signaling pathway, directly binds to the E-box element in *GhBEE3-Like* promoter region and inhibits its expression, which has been confirmed by electrophoretic mobility shift assay (EMSA) and dual luciferase reporter assay. Functional analysis revealed that *Arabidopsis* with *GhBEE3-Like* overexpression had drought sensitive phenotype, while *GhBEE3-Like* knock-down cotton plants obtained by virus-induced gene silencing (VIGS) technology were more tolerant to drought stress. Furthermore, the expression levels of three stress-related genes, *GhERD10*, *GhCDPK1* and *GhRD26*, were significantly higher in *GhBEE3-Like* knock-down cotton than in control cotton after drought treatment. These results suggest that *GhBEE3-Like* is inhibited by BRs which elevates the expressions of stress-related genes to enhance plant drought tolerance. This study lays the foundation for understanding the mechanisms of BR-regulated drought tolerance and establishment of drought-resistant cotton lines.

## Introduction

Brassinosteroids (BRs) are a specific class of steroidal hormones in plants, and were initially found in the pollen of *Brassica napus* (Grove et al., 1979). At present, there are more than 70 compounds of BRs identified in different plant species. BRs play vital roles in regulating multiple physiological processes such as photomorphogenesis, seed germination, root development, cell division and elongation/differentiation, reproductive processes, guard cell development, senescence, and biotic/abiotic stress responses (Tang et al., 2016; Liu et al., 2018a; Ahammed et al., 2020; Lin, 2020; Manghwar et al., 2022; Xiong et al., 2022).

BR signal transduction has been extensively studied in recent years. BRs are perceived by the extracellular domain of BR insensitive-1 (BRI1), a plasmatic membrane receptor-like kinase (RLK) (Shiu et al., 2004). The BRI1 dimerizes with BRI1-associated receptor kinase 1 (BAK1) or somatic embryogenesis receptor kinase 1 (SERK1) after binding with BRs (Li et al., 2002; Russinova et al., 2004). Sequential trans-phosphorylation between BAK1 and BRI1 activates them, and the kinases further phosphorylate the inhibitor of BRI1 (BKI1), leading to its association with 14-3-3 proteins (Wang and Chory, 2006; Jaillais et al., 2011). Concomitantly, the activated BRI1 phosphorylates constitutive differential growth 1 (CDG1) and BR-signaling kinase 1 (BSK1), which both phosphorylate and active BRI1-supressor 1 (BSU1) phosphatase (Mora-García et al., 2004; Tang et al., 2008; Kim and Wang, 2010; Kim et al., 2011). Additionally, BSK3 (BRI1-associated receptor kinase 3) functions as a BR signaling scaffold and upregulates BSU1 protein levels (Ren et al., 2019). BSU1 subsequently dephosphorylates the GSK3-like kinase BR insensitive 2 (BIN2), which is posteriorly inhibited by Kink suppressed in bzr1-1D (KIB1) (Ryu et al., 2010; Zhu et al., 2017). Upon BIN2 inactivation, the two homologous transcription factors (TFs) in BR signaling pathway, bri1-EMS-suppressor 1 (BES1) and brassinazole resistant 1 (BZR1), are dephosphorylated by phosphatase 2 (PP2A) and consequently dissociated from 14-3-3 proteins, resulting in the binding to E-Box (CANNTG) and BRRE elements (CGTG^T^/_C_G) in the promoters of BR-responsive genes (Wang et al., 2006; Li and Jin, 2007; Wang et al., 2014). In the absence of BR, BKI1 can bind to the intracellular domain of BRI1 and prevent its association with BAK1, thus resulting in the inhibition of phosphorylation cascade (Wang and Chory, 2006). In turn, BIN2 may not be inactivated, and 14-3-3 proteins associate with BZR1 and BES1, which retain their dephosphorylation form and inhibit their ability to modulate the expression of BR responsive genes (Jaillais et al., 2011), and stimulate their degradation (Kim et al., 2019).

The basic helix-loop-helix (bHLH) protein family is the second largest family of TFs in plants (Feller et al., 2011). The family is defined by its typical feature, bHLH domain which holds about 60 amino acids, including two diverse regions: the basic region (b region) and the HLH region (Murre et al., 1989a; Jones, 2004). The basic domain located at the N-terminal end is liable for recognizing and binding to the DNA regulatory motif in the promoters of their target genes (Toledo-Ortiz et al., 2003; Li et al., 2006). bHLH proteins are typically divided into two groups based on different functions of the basic region: DNA- and non-DNA-binders (Jones, 2004). The HLH domain at the C-terminus carries two amphipathic α-helices joined by a loop with variable sequences and involves in homo- or heterodimerization with other bHLH proteins (Toledo-Ortiz et al., 2003; Feller et al., 2011). Several reports have mentioned that bHLH proteins are involved in the processes of morphogenesis (De Lucas and Prat, 2014; Li et al., 2016a; Gangappa and Kumar, 2017), iron homeostasis (Li et al., 2016b), light signaling (Leivar et al., 2012), flowering time (Ito et al., 2012), biotic and abiotic stresses (Waseem et al., 2019; Verma et al., 2020), and hormonal signals (Liu et al., 2018b).

A number of bHLH family proteins are involved in BR signaling pathway (Nemhauser et al., 2004; Vert et al., 2005). The reasonable explanation for this may be that BES1 and BZR1, the key TFs in BR signaling pathway, contain bHLH domains, and their recognized elements are found in the promoter of many bHLH genes (Yin et al., 2005). BR Enhanced Expression (BEE) genes, such as *BEE1*, *BEE2* and *BEE3*, are BR early-response genes and have redundant function in BR signaling pathway, which belong to bHLH family genes (Friedrichsen et al., 2002). Here, we identified a bHLH family gene, namely, *GhBEE3-Like*. Quantitative RT-PCR (qPCR), electrophoretic mobility shift assay (EMSA) and dual luciferase reporter assay demonstrated that GhBZR1, the key TF in BR signaling pathway, can bond to the promoter of *GhBEE3-Like* to inhibit its expression. Moreover, the *GhBEE3-Like* transgenic *Arabidopsis* was more sensitive to drought stress, and the cotton with *GhBEE3-Like* gene knock-down by virus-induced gene silencing (VIGS) exhibited drought resistance phenotype. The results of water loss rate, stomatal aperture and the expression of stress-related genes (*GhERD10*, *GhCDPK1* and *GhRD26*) also indicated the similar plant phenotypes under drought stress conditions. Our study reveals the mechanism of BRs repressing the expression level of *GhBEE3-Like* to enhance cotton drought tolerance, which may lay a foundation for the establishment of drought resistance cotton lines.

## Materials and methods

### Plant samples and experimental design

Wild-type *Arabidopsis thaliana*, Col-0 (Columbia-0), was employed as the control. The methods of sowing and cultivating *Arabidopsis*, water loss assay and drought experiments of Col-0 and transgenic plants were performed according to a previous report (Chen et al., 2017a).

CCRI24 cotton (*G. hirsutum* L.) was supplied by the Institute of Cotton Research, Chinese Academy of Agricultural Sciences, and was used as a research material. To detect the gene expression of *GhBEE3-Like*, the samples of root, stem, leaf, petal, anther, sepal, ovules of -1 days post-anthesis (dpa) and 0 dpa, fibers (1, 3, 6, 9, 12, 15, 18, 21, 24, 27 and 30 dpa) were harvested and frozen in liquid nitrogen for RNA isolation. The methods of cotton culture, as well as sample handling and collection were conducted as described previously (Chen et al., 2017a). To clone *GhBEE3-Like* gene, the leaf samples from CCRI24 cotton was used for RNA isolation and cDNA synthesis.

To analyze whether *GhBEE3-Like* gene in cotton can respond to osmotic stress, the specific methods of CCRI24 cotton germination and treatment were performed according to a previous study (Chen et al., 2017a). PEG8000 (10%) was used to simulate drought stress to treat the cotton seedlings.

### Protein domain and phylogenetic analyses

*GhBEE3-Like* (Gh_A12G0489) was obtained through TBLASTN method using the protein sequences of BEE3 (AT1G73830) as query to retrieve gene sequences from the cotton (*G. hirsutum* L.) database (https://www.cottongen.org/). Subsequently, the SMART program (http://smart.embl-heidelberg.de/) was employed to analyze the domains containing in GhBEE3-Like protein. To assess the phylogenetic relationships among GhBEE3-Like and its homologous proteins, the BLAST program in NCBI (http://www.ncbi.nlm.nih.gov/) was applied to search all homologous genes, except *BEE3* gene. The phylogenetic tree was generated in MEGA software (version 7.0) by adopting the neighbor-joining (NJ) algorithm, and the parameter setting was adopted from the previous study (Chen et al., 2021).

### RNA extraction and cDNA synthesis

Total RNA was isolated by RNAprep Pure Plant Kit (TIANGEN, Beijing, China), and cDNA synthesis was performed using PrimeScript^®^ RT Reagent Kit with gDNA Eraser (TaKaRa, Dalian, China). The cDNAs were used for semi-quantitative RT-PCR and qPCR.

### Vector construction

To gain the overexpression vector and GFP vector of *GhBEE3-Like* gene, the full-length coding sequence of *GhBEE3-Like* (738 bp) was amplified using two sets of specific primers, and then ligated into the pMD19-T simple vector (TaKaRa, Dalian, China), respectively. Subsequently, *GhBEE3-Like* was digested with *Bam*HI/*Sac*I and *Sal*I/*Bam*HI enzymes in the pMD19-T-GhBEE3-Like recombination vector (TaKaRa, Dalian, China), respectively. The coding sequence of *GhBEE3-Like* was cloned in the *Bam*HI and *Sac*I sits of p6MYC vector to produce overexpression vector *35S::GhBEE3-Like*, and cloned in the *Sal*I and *Bam*HI sits of pEZR (K)-LC to obtain the GFP vector *35S::GFP-GhBEE3-Like*. The primers used for constructing the above-mentioned vectors were *GhBEE3-Like-OV-F*/*GhBEE3-Like-OV-R* and *GhBEE3-Like-GFP-F*/*GhBEE3-Like-GFP-R* (**Supplementary Table 1**).

*GhBEE3-Like* gene fragment (377 bp) was amplified by PCR technology to produce the VIGS vector of *GhBEE3-Like*, while the above-mentioned *35S::GhBEE3-Like* recombination vector was used as a template. The *GhBEE3-Like* gene fragment was digested with *Xba*I and *Sac*I (TaKaRa, Dalian, China), and cloned into the same sites of TRV2 vector to produce the recombination vector TRV2:GhBEE3-Like. The primer sets *GhBEE3-Like-VIGS-F* and *GhBEE3-Like-VIGS-R* were used for the construction of VIGS vector (**Supplementary Table 1**).

### Subcellular localization analysis

To determine the subcellular localization of GhBEE3-Like, *Agrobacterium tumefaciens*-mediated transient transformation method was used to transform the recombination vector *35S::GFP-GhBEE3-Like* into *Nicotiana benthamiana* leaves (Wroblewski et al., 2005). A confocal laser scanning microscope (ZEISS, LSM 780) was employed to record the green fluorescence images 48 h after transformation.

### Virus-induced gene silencing

To analyze the functions of *GhBEE3-Like* gene under drought stress. VIGS technology was applied to produce TRV2 (control) and TRV2:GhBEE3-Like (*GhBEE3-Like* gene knock-down) cottons according to previous studies (Pang et al., 2013; Chen et al., 2021). In the VIGS technology system, *GhPDS* was used as a marker gene to evaluate the presence of albino phenotype in TRV2:GhPDS cotton after knock-down. Trefoil stage cotton seedlings were water withheld for 1 week and then rewatered for 1 week to calculate the survival rates. Moreover, the trefoil stage cotton leaves were collected for RNA isolation and expression analysis of *GhBEE3-Like* gene.

### Semi-quantitative RT-PCR and quantitative RT-PCR

To examine whether *GhBEE3-Like* is highly expressed in *Arabidopsis*, the leaves of Col-0 and transgenic lines were collected for total RNA extraction and cDNA synthesis. PCR was carried out in a heated-lid thermal cycler (Applied Biosystems, USA) as previously described (Chen et al., 2021). The primers of the internal reference *AtUBQ10* (At4g05320) are listed in **Supplementary Table 1**.

To analyze the tissue expression patterns of *GhBEE3-Like* gene and whether *GhBEE3-Like* can respond to drought stress or BR treatment, the expression levels of *GhBEE3-Like* and stress-related genes were detected using the Applied Biosystems QuantStudio 6 Flex system according to a previous report (Chen et al., 2021). *GhHIS3* gene was employed as an internal reference. The primers used for qPCR are presented in **Supplementary Table 2**.

### Electrophoretic mobility shift assay

The coding region of *GhBZR1* (CotAD_50537) was cloned into the pET30a vector to form GhBZR1-His, in which a His tag was fused into the C-terminal of GhBZR1. The reconstruction vector was transformed into *Escherichia coli* BL21 (DE3). The biotin probe labeled at both ends (3′-end and 5′-end) was prepared (Tsingke Biotechnology Co., Ltd., China). The competitor probe was lacking a biotin label. The ESMA experiment was conducted using the LightShift Chemiluminescent EMSA Kit (Thermo Fisher Scientific, USA). The primer sequences for each probe are shown in **Supplementary Table 1**.

### Dual luciferase reporter assay

To further confirm *GhBEE3-Like* expression regulated by GhBZR1 protein, dual luciferase reporter assay were performed. For transcription activity analysis, the coding region of *GhBZR1* was cloned into the restriction sites of *Kpn*I and *Bam*HI in pCambia1300-35S-3×FLAG vector as an effector, with the empty vector of pCambia1300-35S-3×FLAG as control plasmid. The promoter sequence (1980bp) of *GhBEE3-Like* was inserted into the restriction sites of *Kpn*I and HindIII that upstream of firefly LUC gene in pGreenII 0800-LUC vector to generate a reporter plasmid. REN in the pGreenII 0800-LUC vector under the CaMV35S promoter was used as internal reference (Hellens et al., 2005). The effector plasmid and reporter plasmid were cotransformed into 30 days old tobacco leaves using the *Agrobacterium*-mediated method (Zhou et al., 2018). A dual-LUC reporter assay kit (Catalog No.E1910; Promega, USA) was used to measure LUC and REN activities and the binding efficiency was reported as ratio of LUC to REN.

### Statistical analysis

The qPCR reaction of each sample was performed in triplicate, and three biological replicates were applied. The qPCR results were calculated using the 2^-ΔΔCT^ method. For survival rate analysis of *Arabidopsis* and cotton, three biological replicates consisting of 18 plants and 30 cotton plants were analyzed for each group, respectively, and the survival rate was calculated as the percentage of surviving plants to total plants. For the assay of water loss rate, three biological replicates which consist 3 leaves in each group were used, and the water loss rate was calculated as the percentage of leave water reduction to total weight of leaves at each time point we checked. To analyze the stomatal aperture, 27 stomata in each group were analyzed. For the dual luciferase reporter assay, five biological replicates were applied for analyzing. Error bars in our study were obtained using mean with SD. The significant differences in all experiments were analyzed by Student’s *t*-test.

## Results

### *GhBEE3-Lik*e cloning and phylogenetic analysis

**Figure 1:**
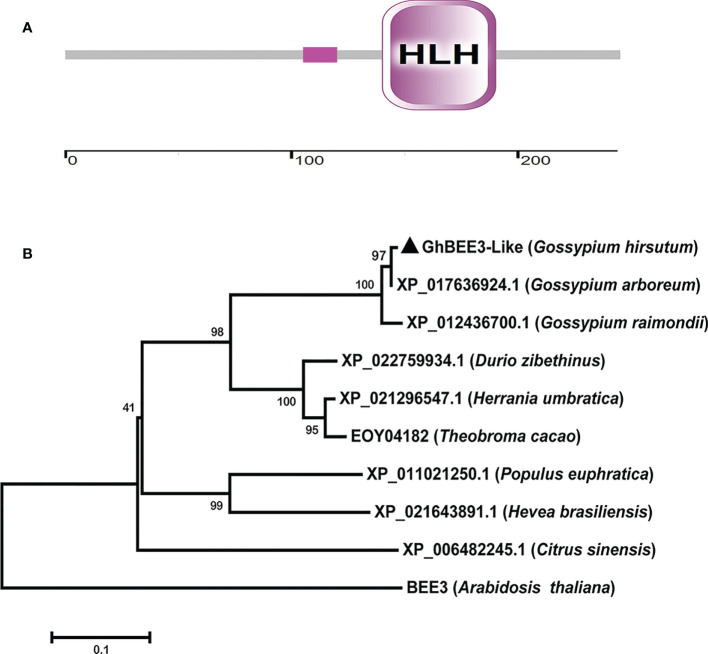
*GhBEE3-Like* protein domain analysis and the phylogenetic relationship between GhBEE3-Like and its homologs in other species. **(A)** Domain analysis of GhBEE3-Like protein. The low compositional complexity is indicated by pink solid rectangle. The ruler is used to measure the number of amino acids. **(B)** The MEGA 7 program was used to generate a phylogenetic tree between GhBEE3-Like and its homologs in other plant species, including *Gossypium arboreum*, *Gossypium raimondii*, *Durio zibethinus*, *Herrania umbratica*, *Theobroma cacao*, *Populus euphratica*, *Hevea brasiliensis*, *Citrus sinensis*, and *Arabidopsis thaliana*. 1,000 replications were used for bootstrap values.

*GhBEE3-Like* sequence was obtained through BLAST program in the *G. hirsutum* L. cotton database (https://www.cottongen.org/) using BEE3 (AT1G73830) protein sequence as query, and then *GhBEE3-Like* was cloned from CCRI24 cotton using a pair of specific primers. Sequence analysis revealed that GhBEE3-Like contained a signature domain, namely, HLH domain (**Figure 1A**). This indicates that GhBEE3-Like is a member of bHLH protein family.

A phylogenetic tree was built according to the protein sequences of GhBEE3-Like and its homologs to analyze their evolutionary relationships. The results showed that GhBEE3-Like clustered together with its paralog XP_017636924.1 in *G. arboreum* (A-genome species) (**Figure 1B**). Moreover, GhBEE3-lik had much closer evolutionary relationship with its ortholog XP_022759934.1 from *Durio zibethinus* (**Figure 1B**). This implies that GhBEE3-Like in *G. hirsutum* (allotetraploid cotton) is originated from XP_017636924.1 in A-genome cotton and share a common ancestor with XP_022759934.1 in *D. zibethinus*.

### *GhBEE3-Like* protein localized in nuclei

**Figure 2:**
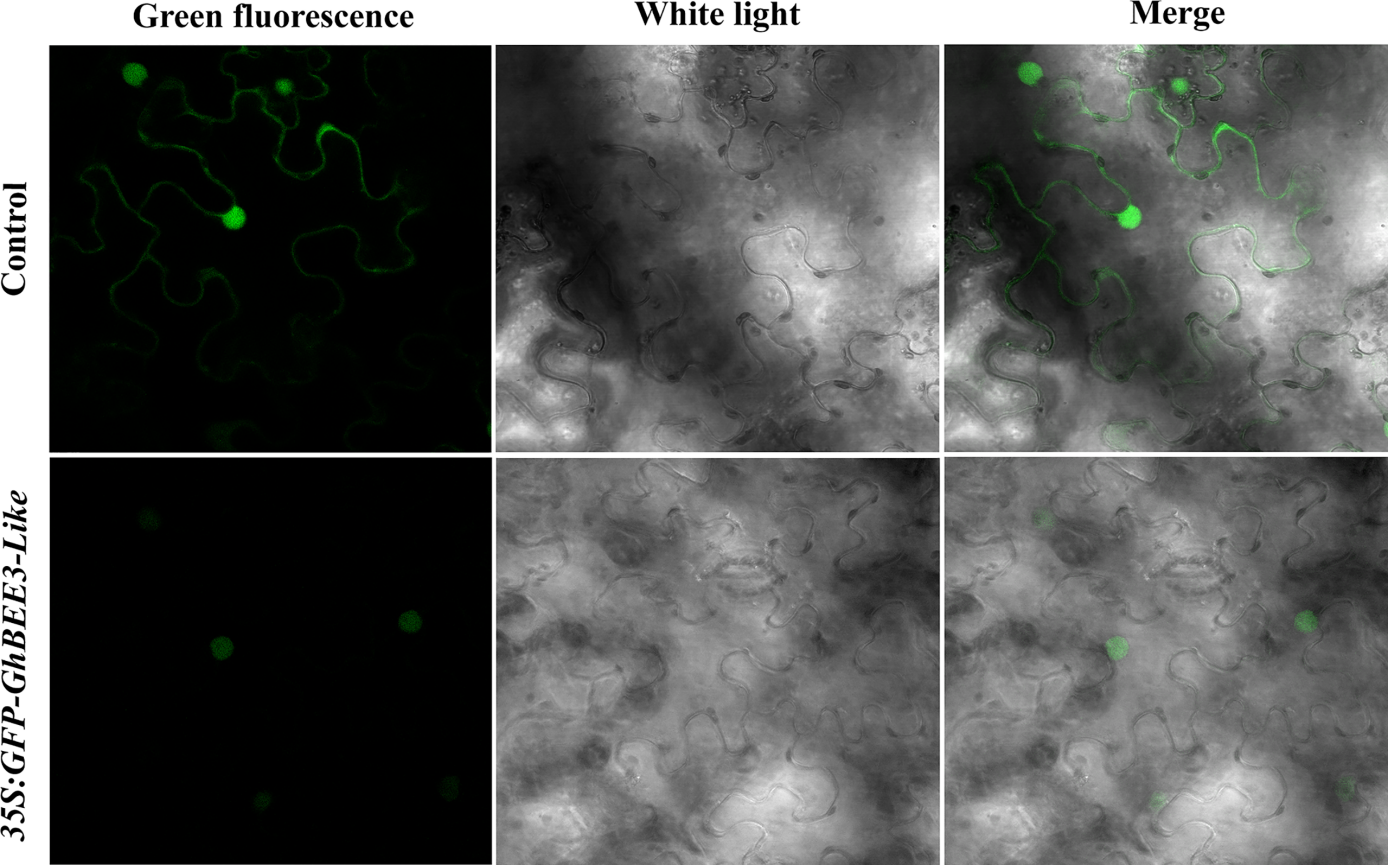
*GhBEE3-Like* protein localization analysis in tobacco mesophyll cells. The three images above indicate the control with free green fluorescence protein (GFP) signals. GFP-GhBEE3-Like fusion protein signals are shown in the three images below. GFP signals indicated that GhBEE3-Like was localized in the nucleus.

To determine the subcellular localization of GhBEE3-Like protein, *GhBEE3-Like* gene was cloned into the C-terminus of a green fluorescent protein (GFP) vector, PEZR(K)-LC. The reconstruction vector was transiently transferred into tobacco leaves and the GhBEE3-Like localization was observed *via* confocal laser scanning microscopy. Unlike the control signals (PEZR(K)-LC vector) that distributed throughout the cells, GFP-GhBEE3-Like was specifically localized in the nucleus (**Figure 2**). These results indicate that GhBEE3-Like can act as a TF due to its localization in the nucleus.

### Expression analysis of *GhBEE3-Like*

**Figure 3:**
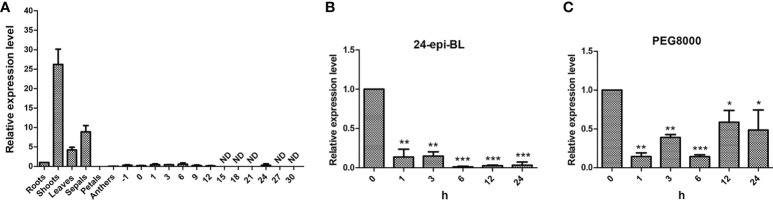
The expression profiling analysis of *GhBEE3-Like* gene. **(A)** Tissue expression analysis of *GhBEE3-Like* in cotton. -1, ovules at -1 days post-anthesis (dpa); 0, ovules at 0 dpa; 1, 3, 6, 9, 12, 15, 18, 21, 24, 27 and 30, fibres at 1dpa, 6 dpa, 9 dpa, 12 dpa, 15 dpa, 18 dpa, 21 dpa, 24 dpa, 27 dpa and 30 dpa. **(B)** The expression analysis of *GhBEE3-Like* in response to 24-epi-BL (an active BR substance). **(C)** The expression analysis of *GhBEE3-Like* in response to PEG8000 (drought simulation). Error bars were conducted with three independent biological replicates. 0 h was used as a control to analyze the expression differences of *GhBEE3-Like* gene at different treated time points. Student’s *t*-test, * P < 0.05, ** P < 0.01, *** P < 0.001.

Determination of gene expression in various tissues helps to understand the gene function in plants. Thus, qPCR was applied to determine the expression patterns of *GhBEE3-Like* in different tissues which include roots, shoots, leaves, sepals, petals, anthers, ovules at -1 dpa and 0 dpa, and fibers at 1 dpa, 3 dpa, 6 dpa, 9 dpa, 12 dpa, 15 dpa, 18 dpa, 21 dpa, 24 dpa, 27 dpa and 30 dpa. The data indicated that *GhBEE3-Like* was highly expressed in shoots, leaves and sepals, especially in shoots, but lowly expressed in other tissues and could not even be detected in fiber samples at 15 dpa (days post-anthesis), 18 dpa, 21 dpa, 27 dpa and 30 dpa (**Figure 3A**). These findings demonstrate that *GhBEE3-Like* can play vital roles in shoot growth and development.

BEE3 is a gene with BR-enhanced expression (Friedrichsen et al., 2002). Thus, BR substance 24-epi-BL was used to treat trefoil-stage cotton seedlings, and the expression profile of *GhBEE3-Like* was detected. *GhBEE3-Like* showed a down-regulated patterns and had significantly lower expression level in 24-epi-BL-treated cotton than that in untreated cotton (0 h), and the lowest expression level occurred at 6 h (**Figure 3B**). These findings indicate that *GhBEE3-Like* is a BRs repressing gene in cotton.

To analyze whether *GhBEE3-Like* can respond to drought stress, 10% PEG8000 was used to treat trefoil-stage cotton seedlings. qPCR data revealed that the expression levels of *GhBEE3-Like* were decreased in the PEG8000-treated samples compared to the untreated sample (0 h) (**Figure 3C**). These results suggest that the expression of *GhBEE3-Like* is repressed by drought stress, and may be involved in drought tolerance.

### GhBZR1 binds to the promoter of *GhBEE3-Like*

**Figure 4:**
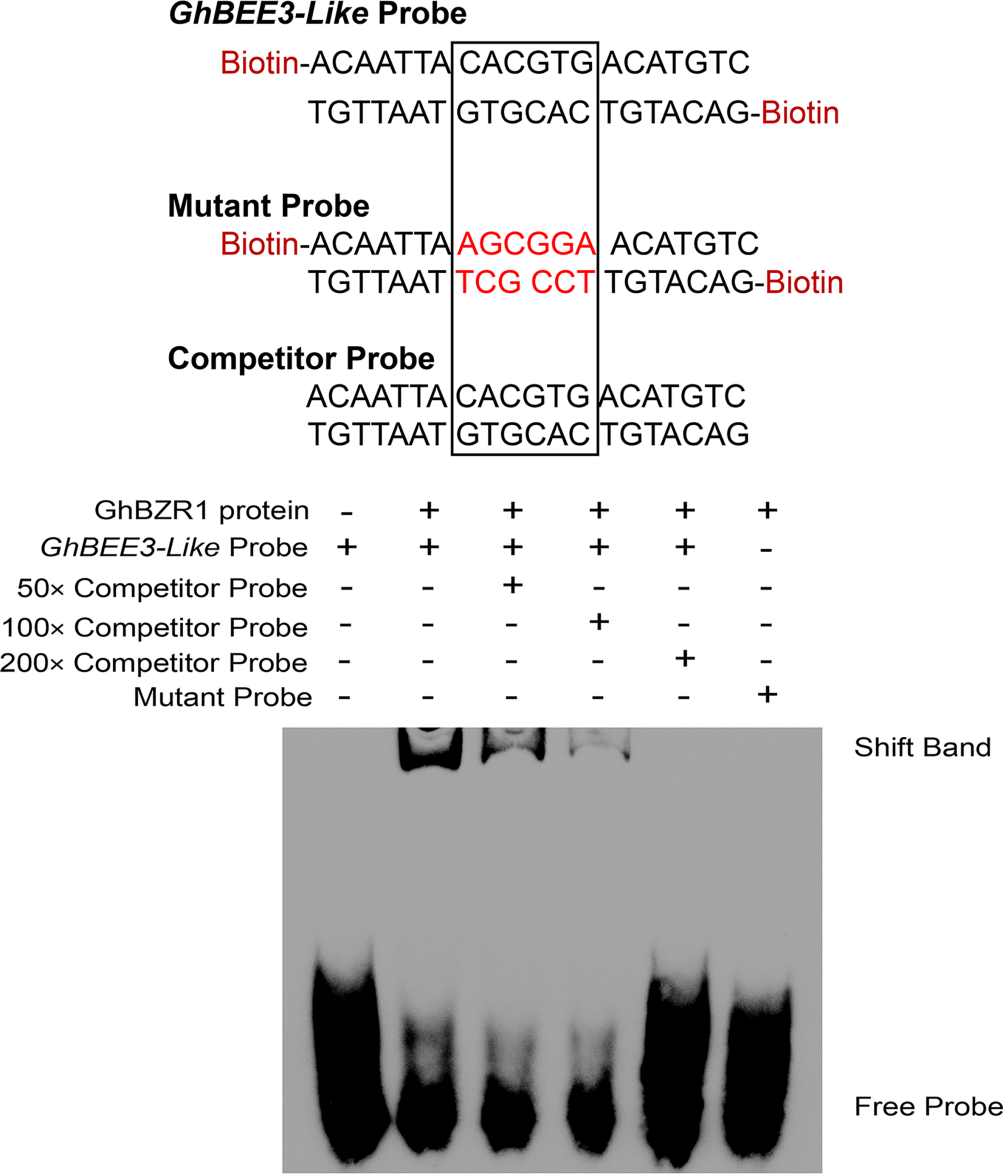
The EMSA (electrophoretic mobility shift assay) displaying the interaction between GhBZR1, a key TF in BR signaling pathway, and the E-box element in *GhBEE3-Like* promoter.

BZR1 is the critical TF of BR signal transduction pathway, which binds to E-box (CANNTG) and BR response elements (BRREs, CGTG^T^/_C_G) in BR-responsive gene promoters to regulate their expression (He et al., 2005; Yin et al., 2005). The EMSA experiment was conducted to explore whether GhBZR1 can bind to the promoter of *GhBEE3-Like*. The results demonstrate that GhBZR1 binds to the biotin probe, *GhBEE3-Like* Probe, which contains an E-box (**Figure 4**). Moreover, the Competitor Probe, which has the same sequences with *GhBEE3-Like* Probe without labeling biotin, could significantly reduce the combination of GhBZR1 protein and *GhBEE3-Like* Probe (**Figure 4**). However, GhBZR1 protein was not bound to the Mutant Probe (**Figure 4**). This indicates that GhBZR1 can bind to the promoter of *GhBEE3-Like* to affect its function in cotton *via* BR signaling pathway.

### GhBZR1 inhibits the expression of *GhBEE3-Like*

**Figure 5:**
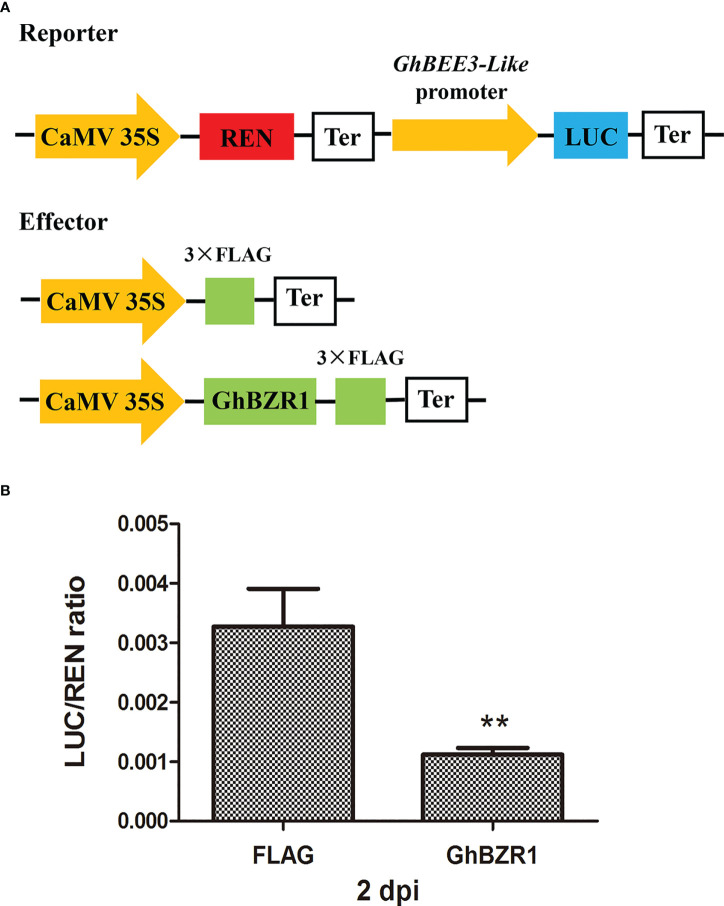
GhBZR1 inhibits the *GhBEE3-Like* expression *in vivo*. **(A)** Schematic model of reporter and effector plasmids used for the dual luciferase report assay. The dual-reporter plasmid contains *GhBEE3-Like* promoter sequences fused to LUC luciferase, and REN driven by CaMV35S promoter was used as internal reference. The effector plasmid contains GhBZR1 or FLAG control. LUC, firefly luciferase; REN, renilla luciferase; Ter, terminator sequence. **(B)** Dual luciferase assay showing the expression inhibition of *GhBEE3-Like* by GhBZR1 TF. The ability of GhBZR1 to regulate the reporter LUC gene was indicated by the ratio of LUC to REN. Asterisks denote significant differences by Student’s *t*-test, **P < 0.01. dpi, days post-inoculation.

To further verify the effect of GhBZR1 on the promoter activity of *GhBEE3-Like*, dual-luciferase (LUC) reporter assay was conducted. The promoter sequences from the upstream start codon of *GhBEE3-Like* were used to drive the firefly LUC gene expression and as a reporter, and the REN in the vector was used as an internal control to normalize the transformation efficiency (**Figure 5A**). GhBZR1 under the CaMV35S promoter was used as the effector, whereas the empty vector with the FLAG labels was served as a negative control (**Figure 5A**). After co-transformation of the GhBZR1effector and reporter in the leaf cells of *N. benthamiana*, the LUC/REN ratio of co-expression of GhBZR1 and *GhBEE3-Like* promoter was significantly lower than the FLAG control at 2 days post-inoculation (dpi) (**Figure 5B**). The results suggest that GhBZR1, the key TF in BR signaling pathway, efficiently inhibits the expression of *GhBEE3-Like*.

### *GhBEE3-Like* gene decreases drought tolerance of transgenic *Arabidopsis*

**Figure 6:**
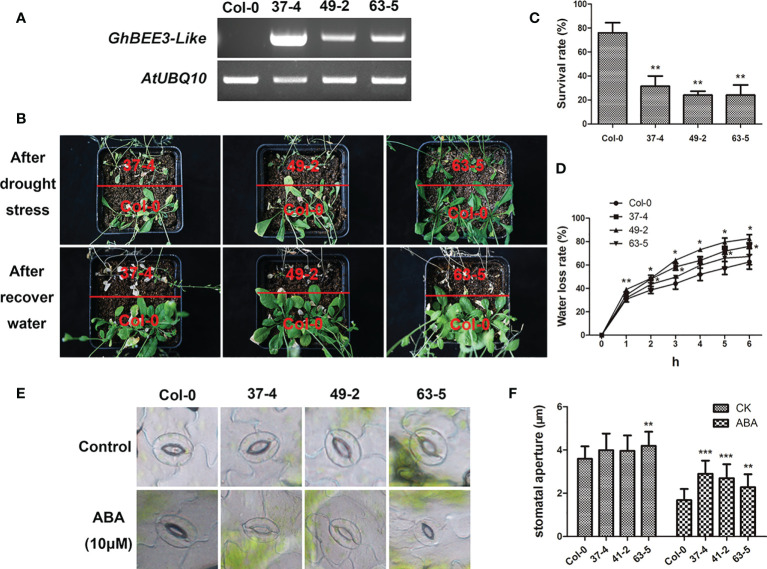
Phenotypic characterization of transgenic *GhBEE3-Like Arabidopsis* under drought conditions. **(A)** Overexpression of *GhBEE3-Like* in *Arabidopsis* was determined by RT-PCR. *AtUBQ10* was employed as an internal reference. **(B)** The phenotypes of Col-0 *Arabidopsis* and *GhBEE3-Like* transgenic lines after drought treatment and water recovery. *GhBEE3-Like* transgenic lines were more vulnerable to drought stress than control plant, Col-0. **(C)** Survival rates of Col-0 *Arabidopsis* and *GhBEE3-Like* transgenic lines after recovery from water loss for 7 days. Three biological replicates were used. Mean ± SD (n = 18, Student’s *t*-test, ** P < 0.01). **(D)** Water loss rates of one-month-old leaves of Col-0 *Arabidopsis* and *GhBEE3-Like* transgenic lines. Three biological replicates were employed for detection. Mean ± SD (n = 3, Student’s *t*-test * P < 0.05, ** P < 0.01). **(E)** Stomata aperture observations of Col-0 *Arabidopsis* and *GhBEE3-Like* transgenic lines under normal conditions and after ABA treatment. **(F)** Statistical analysis of stomata aperture of Col-0 *Arabidopsis* and *GhBEE3-Like* transgenic lines shown in **(E)** Mean ± SD (n = 27, Student’s *t*-test, ** P < 0.01, *** P < 0.001).

BRs are a troop of steroidal hormones that regulate multiple physiological functions, including abiotic stresses. BEE3 is the early response TF that required for BR action. To investigate the function of *GhBEE3-Like* gene on drought stress, the homozygous transgenic *Arabidopsis* plants were chosen and further ensured by semi-quantitative RT-PCR (**Figure 6A**). The transgenic plants and Col-0 plants were grown and withheld water for 3 weeks in the same pot. After water recovery for 1 week, the transgenic lines were more sensitive to drought stress, and the survival rates of *GhBEE3-Like* overexpression lines were remarkably lower compared to Col-0 plant (**Figures 6B,C**). Moreover, the *GhBEE3-Like* overexpression lines showed higher water loss rates compared with Col-0, especially the lines 37-4 and 49-2 (**Figure 6D**). Stomatal aperture is the important factor of plant water loss. The analysis results showed that the transgenic lines had lager stomatal aperture than Col-0 after ABA treatment, and the statistical data were consistent with the phenotypic observation of stomata (**Figures 6E, F**). These findings imply that *GhBEE3-Like* is involved in drought stress and increases the plant sensitivity to drought stress.

### *GhBEE3-Like* knock-down could enhance the cotton tolerance to drought stress

**Figure 7:**
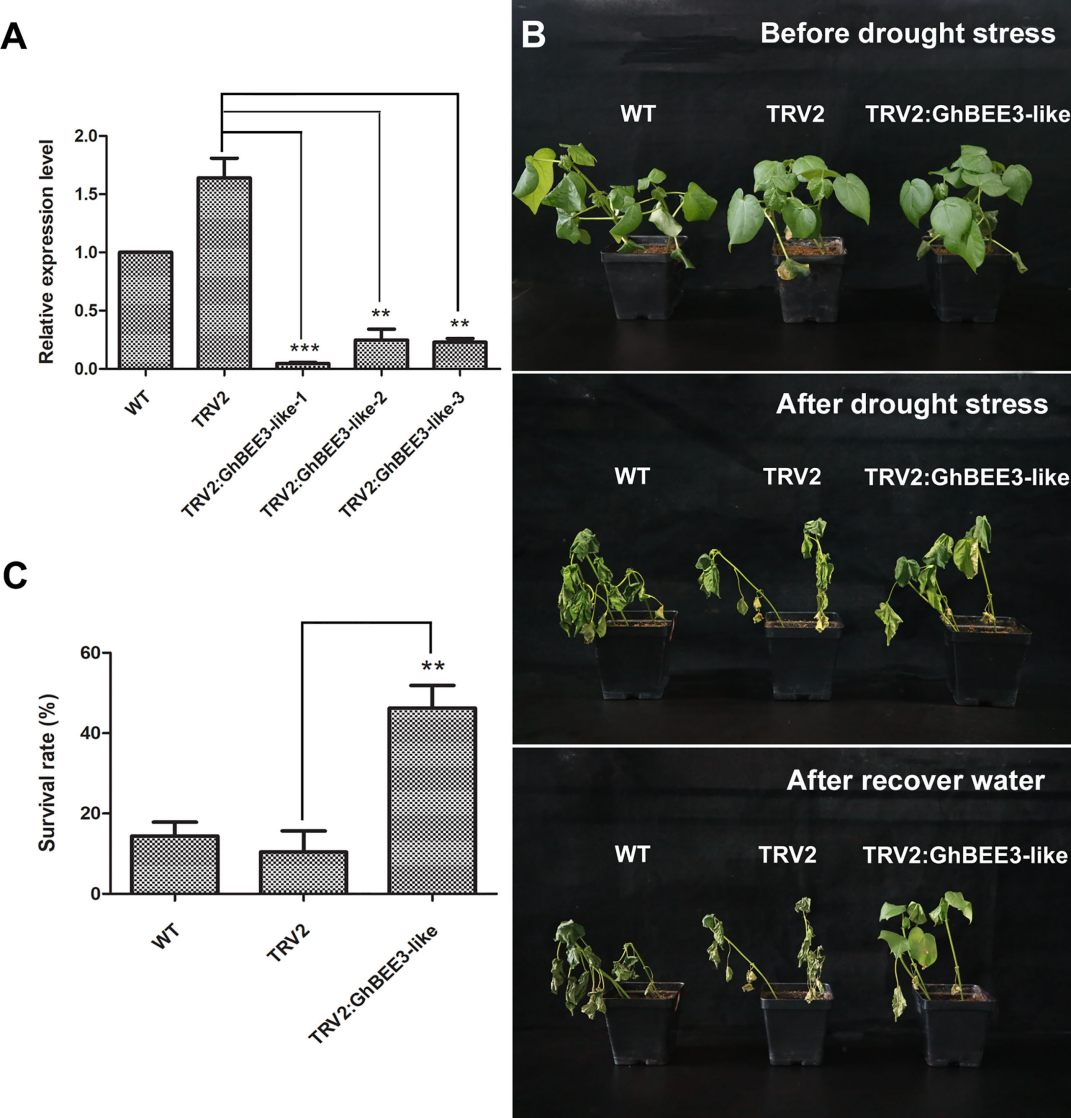
Responses of the WT, TRV2 (TRV2 vector transformation) and TRV2:GhBEE3-Like (*GhBEE3-Like* gene knock-down) cottons to drought stress. **(A)** Expression analysis of *GhBEE3-Like* gene in the three cotton types by qPCR. Significant differences compared to the TRV2 cotton. Student’s *t*-test, ** P <0.01, *** P <0.001. **(B)** Phenotypic observations of the three cotton types before and after drought stress as well as after water recovery. **(C)** Survival rates of the three cotton types at the time of water recovery 7 days after drought stress. Error bars were obtained from three independent biological replicates. Means ± SD (n=30, Student’s *t*-test, ** P <0.01).

To further clarify the function of *GhBEE3-Like* in drought tolerance, VIGS technology was used to knock down *GhBEE3-Like* gene in cotton. Here, *GhPDS* was used as a marker gene to verify the VIGS system on gene knock-down in cottons, and TRV2:GhPDS cotton showed an albino phenotype after two weeks of transformation (**Supplementary Figure 1**). The results demonstrated that VIGS technology could be used for cotton gene knock-down. In addition, the leaves of WT, TRV2 and TRV2:GhBEE3-Like cottons were collected at trefoil stage and qPCR was applied to analyze the knock-down efficiency of *GhBEE3-Like* in WT cotton. Notably, the expression levels of *GhBEE3-Like* in TRV2:GhBEE3-Like cotton plants were significantly lower compared to TRV2 cotton, indicating that *GhBEE3-Like* knocked-down cotton was successfully obtained (**Figure 7A**). Moreover, the WT, TRV2 and TRV2:GhBEE3-Like cottons were withheld water for one week, and then rewatered for growth. One week later, the TRV2:GhBEE3-Like cotton showed more tolerance against drought stress than the WT and TRV2 cottons, and the statistical data of survival rates were consistent with the phenotypic findings (**Figures 7B, C**). These results indicate that *GhBEE3-Like* negatively regulates drought tolerance in cotton.

### Expression analysis of stress-related genes in cotton with *GhBEE3-Like* gene knock-down

**Figure 8:**
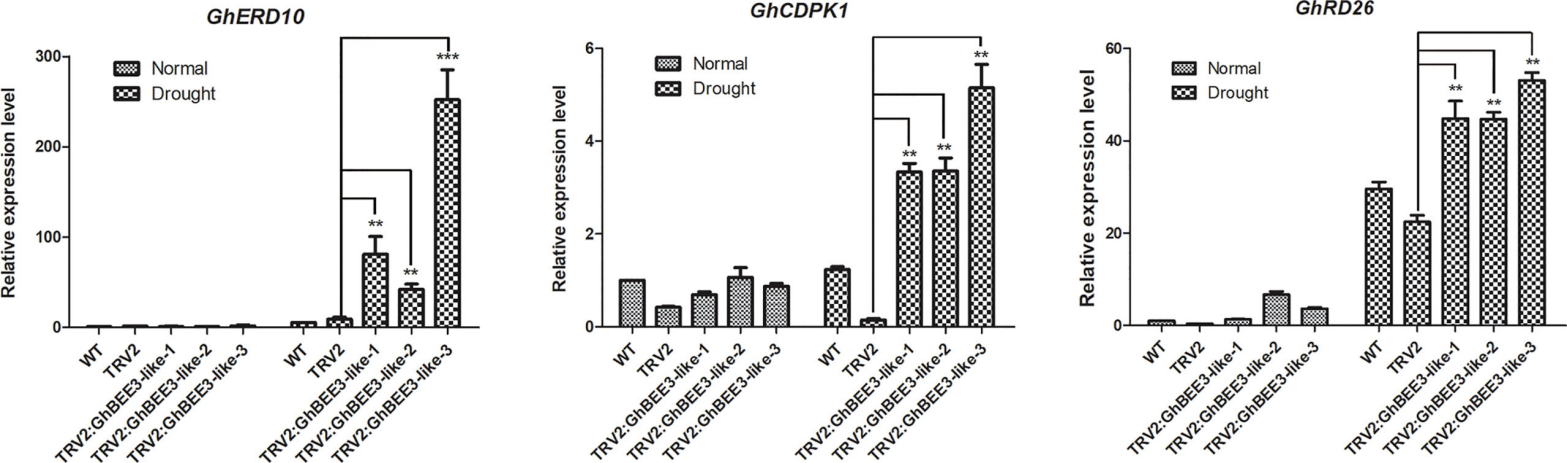
Expression analysis of stress-related genes in the WT cotton (CCRI24), TRV2 cotton (Control cotton) and TRV2:GhBEE3-Like cotton (*GhBEE3* knock-down cotton). The expression levels of stress-related marker genes, *GhERD10*, *GhCDPK1* and *GhRD26*, were significantly higher in the TRV2:GhBEE3-Like cotton than in TRV2 cotton under drought conditions. Error bars represent the SD of three biological replicates. Student’s *t*-test, ** P<0.01, *** P<0.001.

GhBEE3-Like is a TF member of bHLH protein family, which can affect the expression levels of many genes. To further comprehend the molecular control of *GhBEE3-Like* on drought stress, the expression profiles of *GhERD10*, *GhCDPK1* and *GhRD26* were analyzed by qPCR. Under normal conditions, no obvious differences in the expression levels of these three genes were found among the WT, TRV2 and TRV2:GhBEE3-Like cottons. However, these three stress-related genes showed higher expression levels in the TRV2:GhBEE3-Like cotton than in the TRV2 cotton under drought conditions (**Figure 8**). What’s more, the expression levels of these three genes in TRV2:GhBEE3-Like cotton were also higher than those in WT cotton (**Figure 8**). These findings indicate that *GhBEE3-Like* affects many stress-related genes to regulate drought tolerance in cotton.

## Discussion

The bHLH TF family of genes is one of the largest families in eukaryotes and plays significant roles in plant developmental and physiological processes (Ledent and Vervoort, 2001; Liu et al., 2015). The bHLH proteins comprise a basic region (b) followed by a helice-loop-helice (HLH) domain (Ferré-D’amaré et al., 1993). The basic region functions for DNA binding and the HLH domain promotes dimerization with other bHLH proteins (Murre et al., 1989b; Ferré-D’amaré et al., 1993). In upland cotton (*Gossypium hirsutum*) genome, 437 bHLH proteins were identified (Lu et al., 2018). In this study, we cloned a gene, namely, *GhBEE3-Like*. Domain analysis revealed that GhBEE3-Like possessed a typical HLH domain (**Figure 1A**). Subcellular localization analysis revealed that GhBEE3-Like was localized in the nucleus (**Figure 2**). These results indicate that *GhBEE3-Like* is a member of bHLH TF family.

*BEE* genes (*BEE1*, *BEE2*, and *BEE3*) are BR early-response genes and their expression levels were strongly induced by BRs (Friedrichsen et al., 2002). The findings are different with our study in the respect that the expression of *GhBEE3-Like* is decreased after BR treatment (**Figure 3B**). The reason for this may be that different concentrations of BL (an active BR substance) and different culture media were used to treat different plant species. Many studies on the function of *BEE* genes in plants have been reported. Mutations in one or two *BEE1* genes could not result in a distinct phenotype (Friedrichsen et al., 2002). However, *bee1/bee2/bee3*, triple mutants of these genes, showed the typical phenotypes of BR mutants regardless of at seedling or floral stage (Friedrichsen et al., 2002). BEE1 interacts with another bHLH protein CESTA to regulate *CPD*, a BR biosynthesis gene, for activating the biosynthesis of BR in plants through binding to the G-box motif in *CPD* promoter (Poppenberger et al., 2011). In *Arabidopsis*, BEE could affect plant shade-avoidance syndrome by forming a network with BES1-interacting Myc-like1 (BIM) and phytochrome rapidly regulated 1 (PAR1) (Cifuentes-Esquivel et al., 2013). Moreover, inhibiting the activities of *BEE* and *BIM* results in a defect of shade avoidance and a dwarf rosette phenotype (Cifuentes-Esquivel et al., 2013). *BEE1*, *BEE3* and *HAF* (*HALF FILLED*) have similar expression patterns in the reproductive organs, and their triple mutant has defective pollen tube growth (Crawford and Yanofsky, 2011). In cotton, the uncontrolled expression of *GhBEE1-Like* could result in anther indehiscence (Chen et al., 2017b). *PagBEE3L*, a *BEE3-Like* gene in poplar, enhances the proliferation of xylem cells in stems to promote biomass production (Noh et al., 2015). The *bee1*/*bee2* double mutant and *bee1*/*bee2*/*bee3* triple mutant were used to assess the tolerance to drought stress, and the data showed that *bee1*/*bee2*/*bee3* mutant plants were more tolerant to drought stress than Col-0 plants, but the survival rates between *bee1*/*bee2* double mutant and Col-0 had no significant difference (Moreno et al., 2018). These findings imply that *BEE3* may play a vital role on drought resistance in plants. Here, we found that transgenic *Arabidopsis* overexpressing *GhBEE3-Like* were more vulnerable to drought stress than the control plant, Col-0, and the statistical results of survival rates were consistent with the phenotypic findings (**Figures 6A–C**). Furthermore, the water loss rates of *GhBEE3-Like* transgenic *Arabidopsis* lines were relatively higher compared to Col-0 plants, and the stomatal aperture of Col-0 was smaller than that of *GhBEE3-Like* transgenic *Arabidopsis* (**Figures 6D–F**). Additionally, *GhBEE3-Like* gene knock-down cotton obtained by VIGS technology had drought resistant phenotype (**Figure 7**). These results indicate that *GhBEE3-Like* can act as a negative regulator in cotton drought tolerance.

**Figure 9:**
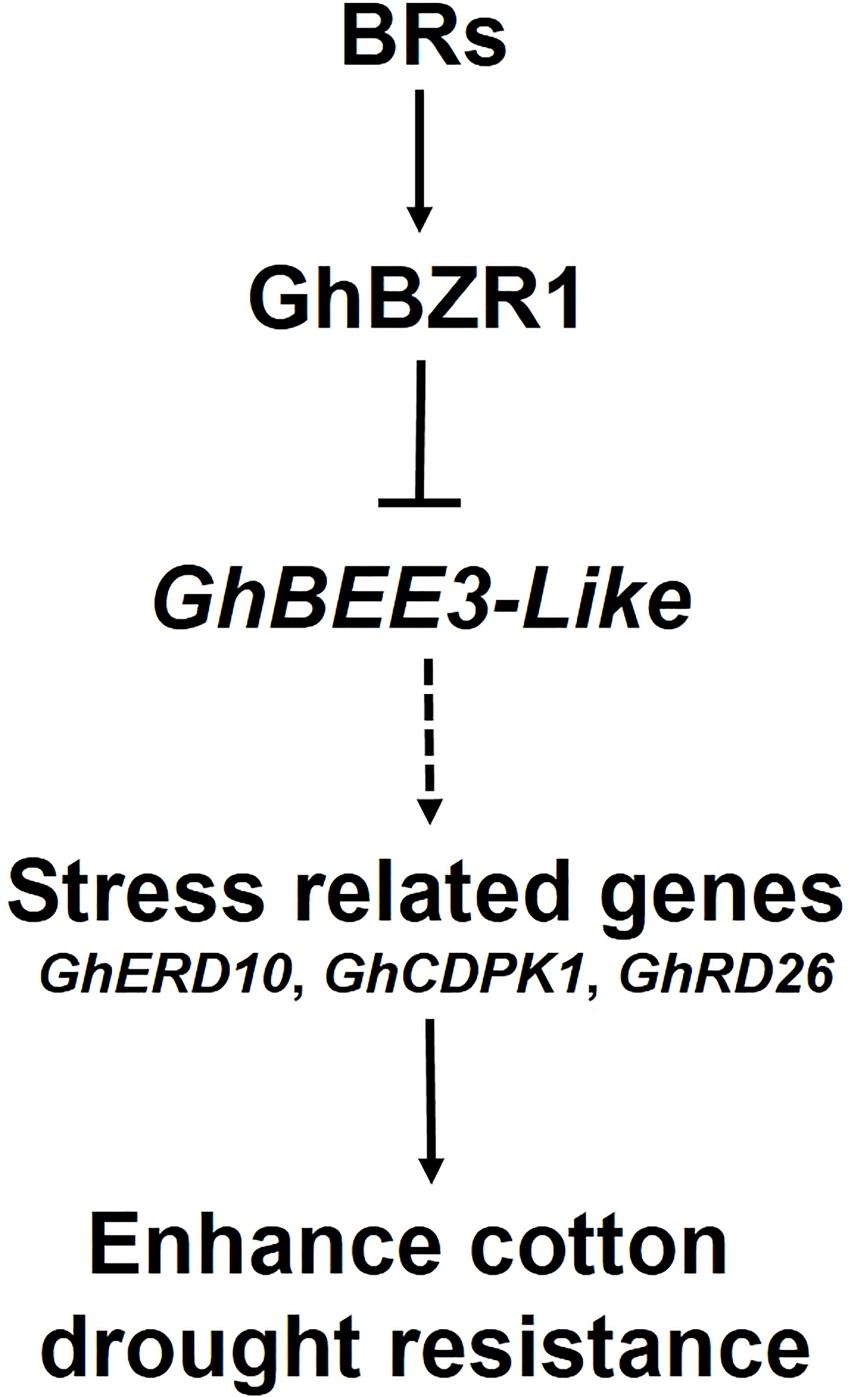
Hypothetical model of BR-regulated drought tolerance in cotton *via GhBEE3-Like*. BRs active GhBZR1, a key TF of BR signaling pathway, to repress the expression of *GhBEE3-Like*. In turn, the down-regulation of *GhBEE3-Like* leads to the high expression of stress-related marker genes that enhance cotton drought tolerance.

BRs, the sixth class of phytohormones, play vital roles in regulating multiple physiological processes, including biotic and abiotic stress responses (Peres et al., 2019). BR signal transduction pathway is that BRs are perceived and bound by the plasmatic membrane receptor-like kinase BRI1 (Shiu et al., 2004). Through a series of transmembrane signal transduction process to activate the key TFs in BR signaling pathway, BES1 and BZR1, to regulate the functions of BR responsive genes (Jaillais et al., 2011). The bHLH proteins are involved in various biological processes, including drought and osmotic stress signaling (Liu et al., 2014) and BR signaling pathway (Zhang et al., 2009). *BEE3* is a bHLH family gene and responses to BRs at early stage (Friedrichsen et al., 2002). In previous study, our results found that BR deficiency caused higher sensitivity to drought stress in cotton (Chen et al., 2019). So, does BRs regulate *BEE3* gene to affect plant drought tolerance? Here, *GhBEE3-Like*, a bHLH TF family gene in cotton, is repressed by BRs at transcriptional level (**Figures 1**-**3**). EMSA results demonstrated that GhBZR1, the key TF in BR signaling pathway, could bind to the E-box motif in *GhBEE3-Like* gene promoter (**Figure 4**). Additionally, the results of dual-luciferase reporter assay showed that GhBZR1 repressed the gene expression of *GhBEE3-Like* (**Figure 5**). All the results confirmed that *GhBEE3-Like* was a BR-regulated gene in cotton. Moreover, *Arabidopsis* with *GhBEE3-Like* overexpression were more sensitive to drought stress, and knocking down the *GhBEE3-Like* gene in cotton with VIGS technology could enhance cotton drought tolerance and induce the expression of many stress-related genes (*GhERD10*, *GhCDPK1* and *GhRD26*) under drought conditions (**Figures 6**–**8**). Hence, we speculate that a regulatory mechanism is involved in cotton drought resistance by which BRs active GhBZR1 to repress the expression of *GhBEE3-Like*, which in turn induces the expression of stress-related genes to enhance cotton resistance under drought stress (**Figure 9**).

Overall, we propose a molecular mechanism of cotton drought resistance regulated by *GhBEE3-Like* gene *via* BR signaling pathway. *GhBEE3-Like*, a bHLH TF family gene, is repressed by the key TF of BR signaling pathway, GhBZR1. Moreover, transgenic *Arabidopsis* overexpressing *GhBEE3-Like* were more sensitive to drought stress, and knocking down *GhBEE3-Like* gene with VIGS technology could enhance cotton drought resistance. The expression levels of three stress-related genes, *GhERD10*, *GhCDPK1* and *GhRD26*, were upregulated in cotton plants after *GhBEE3-Like* knock-down under drought conditions. These results deepen our understanding of BR-regulated mechanisms on cotton drought resistance mediated by *GhBEE3-Like*.

## Data availability statement

The original contributions presented in the study are included in the article/**Supplementary Material**. Further inquiries can be directed to the corresponding authors.

## Author contributions

CL and HH conceived and designed the research; EC and XY performed the experiments; RL, MKZ, and MZ analyzed the data; FZ and DL read and provided helpful discussion; EC wrote the original draft; CL and HH reviewed and edited the manuscript. All authors read and approved to publish the final manuscript.

## Funding

This research was supported by the National Natural Science Foundation of China (Grant No. 32101679), the Natural Science Foundation of Henan Province (Grant No. 202300410162), the Doctoral Research Start-up Fund of Henan Institute of Science and Technology (Grant No. 2017006) and the National Natural Science Foundation of China (Grant No. 31900394).

## Conflict of interest

The authors declare that the research was conducted in the absence of any commercial or financial relationships that could be construed as a potential conflict of interest.

## Publisher’s note

All claims expressed in this article are solely those of the authors and do not necessarily represent those of their affiliated organizations, or those of the publisher, the editors and the reviewers. Any product that may be evaluated in this article, or claim that may be made by its manufacturer, is not guaranteed or endorsed by the publisher.
