# Supplementary material for: GhBEE3-Like gene regulated by brassinosteroids is involved in cotton drought tolerance
Source: Front Plant Sci. 2022 Oct 13;13:1019146. doi: 10.3389/fpls.2022.1019146 (PMC9606830; doi:10.3389/fpls.2022.1019146)
Supplement: Supplementary file 1 [file DataSheet_1.docx]

Supplementary Material

# Supplementary Figures and Tables

## Supplementary Figures


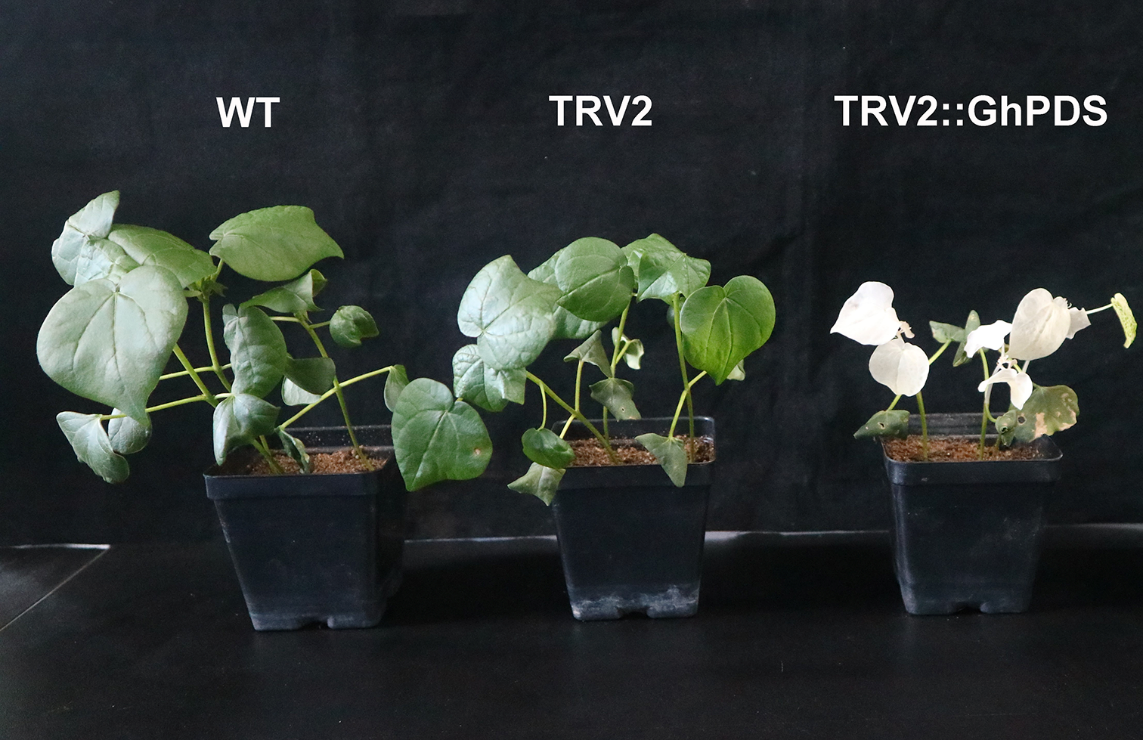


**Supplementary Figure 1.** Phenotype of *GhPDS* knock-down cotton (TRV2:GhPDS cotton) using the VIGS technology.

## Supplementary Tables

**Supplementary Table 1** Primers used in this study.

| **Primer name** | **Primer** |
| --- | --- |
| *GhBEE3-Like-OV-F* | GGATCCTAATGGGTGAGTTTAGACAACAT |
| *GhBEE3-Like-OV-R* | GAGCTCTCAAAGTGGCCATGTCGAAT |
| *GhBEE3-Like-GFP-F* | GTCGACAGATGGGTGAGTTTAGACAACAT |
| *GhBEE3-Like-GFP-R* | GGATCCTCAAAGTGGCCATGTCGAAT |
| *GhBEE3-Like-VIGS-F* | TCTAGA ATGGGTGAGTTTAGACAACAT |
| *GhBEE3-Like-VIGS-R* | GAGCTCTGATTTCCTCTGCTTCTTTTG |
| *AtUBQ10-F* | GATCTTTGCCGGAAAACAATTGG |
| *AtUBQ10-R* | TAGAAAGAAAGAGATAACAGG |
| *GhBEE3-Like Probe-F* | Biotin-ACAATTACACGTGACATGTC |
| *GhBEE3-Like Probe-R* | TGTTAATGTGCACTGTACAG-Biotin |
| *Mutant Probe-F* | Biotin-ACAATTAAGCGGAACATGTC |
| *Mutant Probe-R* | TGTTAATTCGCCTTGTACAG-Biotin |
| *Competitor Probe-F* | ACAATTACACGTGACATGTC |
| *Competitor Probe-R* | TGTTAATGTGCACTGTACAG |
| *GhBEE3-Like-LUC-F* | ctatagggcgaattgggtaccGAGTGGGGAACATGTCGTGAC |
| *GhBEE3-Like-LUC-R* | caggaattcgatatcaagcttATTGGAAGAAGGATGTTCTAA |
| *GhBZR1-FLAG-F* | catttggagaggacagggtaccATGACGTCAGATGGGGCGACG |
| *GhBZR1-FLAG-R* | ctagtgtcgactctagaggatccTCAACATCGAGCTTTCCCACT |

**Supplementary Table 2** The qRT-PCR primers used for gene expression analysis of *GhBEE3-Like* and stress related genes.

| **Primer name** | **Sense primer** | **Antisense primer** |
| --- | --- | --- |
| *GhBEE3-Like* | AGCAGTGTTCAGAGTTGAATC | TCCGTTGCTTTTCTCTTGTTG |
| *GhERD10* | CTTTCTCAGAGAACGGGTTC | GCTGGTGGTTGGGTGGTG |
| *GhCDPK1* | CTGTAACGCTTGCGTTAGAC | CTTTCTCAGAGAACGGGTTC |
| *GhRD26* | GTGTTTCAAGCAGAGAGCAA | AAATTCCCGATACCCAGACTT |
| *GhHIS3* | GCCAAGCGTGTCACAATTATG | ACATCACATTGAACCTACCACTACC |
